# Clinical guidance for podiatrists in the management of foot problems in rheumatic disorders: evaluation of an educational programme for podiatrists using a mixed methods design

**DOI:** 10.1186/s13047-020-00435-7

**Published:** 2021-02-25

**Authors:** E. J. Huijbrechts, J. Dekker, M. Tenten-Diepenmaat, M. Gerritsen, M. van der Leeden

**Affiliations:** 1 Amsterdam Rehabilitation Research Centre | Reade, Dr. Jan van Breemenstraat 2, PO 58271, 1040 HG Amsterdam, The Netherlands; 2 Fontys University of applied sciences | Department of allied health professionals, Fontys Paramedische Hogeschool, Eindhoven, The Netherlands; 3 Department of Rehabilitation Medicine, Amsterdam UMC, Vrije Universiteit van Amsterdam, Amsterdam, The Netherlands; 4 Saxion University of applied sciences | department of healthcare, Saxion, Enschede, The Netherlands

**Keywords:** Rheumatic disorders, Foot problems, Podiatry, Pilot

## Abstract

**Background:**

Foot and ankle problems are common in rheumatic disorders and often lead to pain and limitations in functioning, affecting quality of life. There appears to be large variability in the management of foot problems in rheumatic disorders across podiatrists. To increase uniformity and quality of podiatry care for rheumatoid arthritis (RA), osteoarthritis (OA), spondyloarthritis (SpA), and gout a clinical protocol has been developed.

**Research objectives:**

[1] to evaluate an educational programme to train podiatrists in the use of the protocol and [2] to explore barriers and facilitators for the use of the protocol in daily practice.

**Method:**

This study used a mixed method design and included 32 podiatrists in the Netherlands. An educational programme was developed and provided to train the podiatrists in the use of the protocol. They thereafter received a digital questionnaire to evaluate the educational programme. Subsequently, podiatrists used the protocol for three months in their practice. Facilitators and barriers that they experienced in the use of the protocol were determined by a questionnaire. Semi-structured interviews were held to get more in-depth understanding.

**Results:**

The mean satisfaction with the educational programme was 7.6 (SD 1.11), on a 11 point scale. Practical knowledge on joint palpation, programme variation and the use of practice cases were valued most. The protocol appeared to provide support in the diagnosis, treatment and evaluation of foot problems in rheumatic disorders and the treatment recommendations were clear and understandable. The main barrier for use of the protocol was time. The protocol has not yet been implemented in the electronic patient file, which makes it more time consuming. Other experienced barriers were the reimbursement for the treatment and financial compensation.

**Conclusions:**

The educational programme concerning the clinical protocol for foot problems in rheumatic disorders appears to be helpful for podiatrists. Podiatrists perceived the protocol as being supportive during patient management. Barriers for use of the protocol were identified and should be addressed prior to large scale implementation. Whether the protocol is also beneficial for patients, needs to be determined in future research.

## Background

Foot and ankle problems are highly prevalent in rheumatic conditions such as rheumatoid arthritis (RA) [1–4], osteoarthritis (OA) [5], gout [6] and spondyloarthritis (SpA) [7–9]. Impairments of the foot and ankle as a result of these rheumatic conditions can lead to pain, limitations in daily functioning [5, 6, 10, 11] and reduced quality of life [12–14]. Due to the complexity of rheumatic foot problems, a multidisciplinary approach to management is crucial [15].

Multidisciplinary recommendations for the diagnosis and treatment of foot problems in people with rheumatoid arthritis (RA) have recently been published [16], with levels of evidence assigned to every recommendation [17]. According to these recommendations, podiatrists have an important role in the management of rheumatic foot problems, especially with regard to biomechanical and dermatological impairments. Treatment by podiatrists can include information and education of patients on foot health and shoe advice, treatment with foot orthoses, silicon toe orthoses, foot exercises or wound care [16]. Specific guidance for podiatrists was not provided in the multidisciplinary recommendations. Therefore, as a next step, a clinical protocol for podiatry management of foot problems in rheumatic disorders has been developed in the Netherlands, based on literature review and opinions from experts (a summary of the protocol can be found in Methods section) [18]. Clinical guidance by the use of a protocol can decrease the known variability in podiatry care and it can increase the quality of care [19]. However, application of such a protocol requires specific knowledge and skills of podiatrists.

These competencies seem underdeveloped because postgraduate rheumatology education for podiatrists is lacking in the Netherlands, as seems to be the case in most European countries. Based on results of a survey about educational needs of health professionals, postgraduate rheumatology education was most common for nurses, physical and occupational therapists [20]. The lack of specialized podiatrists in rheumatology is supported by a study of Williams et al. [21], in which the use of management guidelines for foot problems in rheumatoid arthritis in the United Kingdom was investigated. In that study only 16 out of the 245 podiatrists (6,5%) who responded to the survey were specialists in rheumatology. Of the non-specialist podiatrists (93,5%), 97% were unaware of the guidelines. The importance of training of clinicians, but also the unmet needs of patients with rheumatic disorders, was stressed in other studies as well [22–24].

Education of podiatrists seems therefore crucial at this stage of implementation of the clinical protocol for podiatry management of foot problems in rheumatic disorders. At the same time, barriers and facilitators for use of the protocol need to be evaluated, prior to large-scale implementation. Therefore, an educational programme was developed to train podiatrists in the use of the protocol in their daily clinical practice.

The aims of this study were: 1) to evaluate an educational programme to train podiatrists in the use of the protocol and 2) to explore barriers and facilitators to the use of the protocol in daily practice.

## Method

### Study design

A mixed method design was used to evaluate the educational programme as well as exploring the barriers and facilitators for use of the protocol in podiatry practice. A quantitative analysis of questionnaires was combined with a qualitative analysis of semi-structured interviews.

An educational programme was developed to educate podiatrists in the use of the systematic podiatry protocol. After the educational programme, participants were asked to fill out a questionnaire to evaluate the programme. Podiatrists then used the protocol three months in daily practise. They received several tools that they could use, such as a digital checklist with all items of the protocol and a checklist on paper [25]. Both the digital checklist and the checklist on paper consists of eight sections of the podiatrists’ diagnostic phase: 1) patient history, 2) inspection, 3) palpation, 4) function tests, 5) additional testing, 6) gait analysis, 7) pressure measurements and 8) shoe inspection. Each section contains the point of attention for the podiatrists with regard to foot problems in patients with rheumatic disorders. Coaching on the use of the protocol was available during the pilot phase by the researcher (EHUI), if necessary. The number and duration of the coaching session was not prescribed. It was recommended that podiatrists contacted - by telephone or email - the researcher (EHUI) if they had questions about the content of the protocol or if they had substantive questions about foot problems in patients with a rheumatic disorder. After the pilot phase, participants were asked to fill out a digital questionnaire on the facilitators and barriers in the use of the protocol. In addition, podiatrists were asked to participate in semi-structured interviews to gain more in-depth understanding of the facilitators and barriers.

### Participants

Podiatrists could register via a request from the Dutch Association of Podiatrists (NVvP). The NVvP is a professional association with about 1000 podiatrists and a coverage ratio of almost 100% in the Netherlands. The NVvP selected 35 graduated podiatrists from small, medium and large practices (based on the number of branches of the practice), different ages and different levels of experience, throughout the Netherlands. By far the majority of podiatrists in the Netherlands work in private practices. A minority is employed by a hospital; some podiatrists hold consultations from their private practice in a specific department of a hospital (such as internal medicine, orthopaedics, rehabilitation or rheumatology). In the Netherlands, patients with foot problems in rheumatic disorders can visit the podiatrist without referral or they can be referred by a medical doctor. Reimbursement of podiatry care in patients with foot problems in rheumatic disorders comes from the patient’s supplementary insurance, with a cap on the amount.

A power analysis was not applicable due to the nature of the study. The choice of a sample size of 35 was based on practical reasons and as well on the sample needed to reach data saturation in the qualitative analysis. The Medical Ethics Review Committee of VU University Medical Centre, the Netherlands, confirmed that the Medical Research Involving Human Subjects Act (WMO) did not apply to this study (see Additional file 1). All participants gave a written informed consent.

### The clinical protocol for podiatry management in rheumatic foot problems

**Fig. 1:**
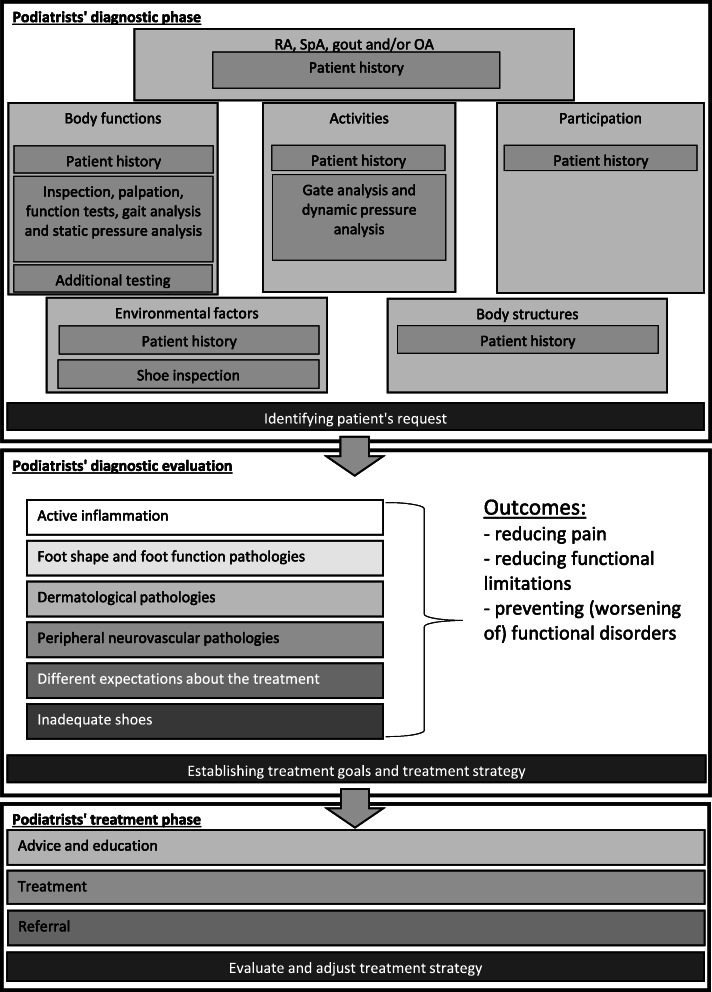
The podiatry protocol for rheumatic foot problems

The protocol applies to four common rheumatic disorders: 1) RA, 2) SpA, 3) gout and 4) OA. Six diagnostic categories can be distinguished: 1) active inflammation of foot structures, 2) abnormal foot shape and foot function pathologies, 3) dermatological pathologies, 4) peripheral neurovascular pathologies, 5) different expectations about the treatment and 6) inadequate shoes. One or more categories can be applicable per patient. For every diagnostic category, the protocol describes several treatment options, including patient advice and education, podiatry treatment and referral to other health care providers, based on best available evidence (see also Fig. 1) [25]. A summary of the development of the protocol can be found in Additional file 2.

**Fig. 2:**
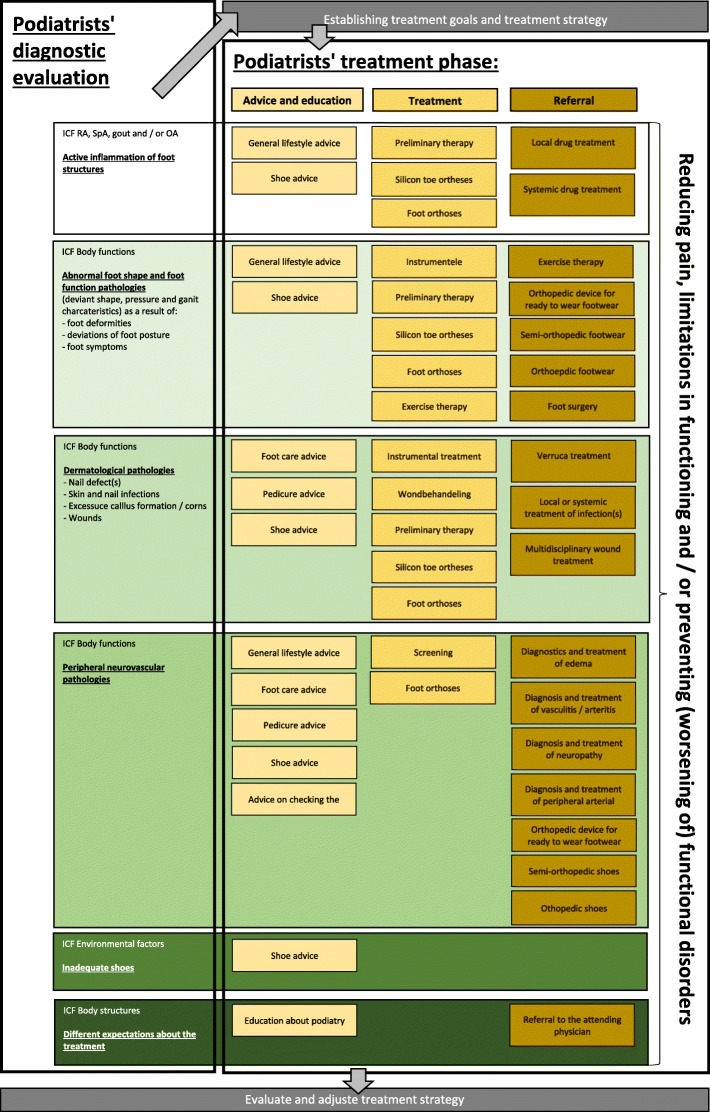
Different treatment options per diagnostic category

Examples of patient advice and education are: general lifestyle advice, shoe advice or education about foot health. Silicon toe orthoses, foot orthoses and wound care are examples of podiatry treatment. Examples of referral to other health care providers are: referral for local or systemic drug treatment of inflammatory activity, referral for orthopaedic shoes or referral for foot surgery. A more detailed description of treatment options per diagnostic category can be found in Fig. 2.

### Educational programme

Lecturer/podiatrist/researcher (EHUI) developed the educational programme. A blended learning method to develop the educational programme was used [26]. This method uses face-to-face activities mixed with computer-mediated activities (such as digital and online activities). Topics of the educational programme where discussed in a consensus meeting with 11 podiatrists, held during the development of the protocol. Seven participants of the consensus meeting also participated in the evaluation of the protocol. The educational programme consisted of a day program of six hours with lectures and workshops and an e-learning of 30 min with regard to: 1) theory on foot problems in rheumatic disorders, 2) theory on dermatological foot problems in rheumatic disorders, 3) a workshop in the use of ultrasound and palpation of foot synovitis and 4) practical examples of diagnosis and treatment of foot problems in rheumatic disorders. The programme started with a lecture of an hour on new insights in rheumatic disorders, medication use and its relevance for foot complaints, different treatment options and communication with doctors. In the following hour, the use of the protocol was introduced and in case study presentations participants practiced the use of the protocol and discussed the recognition of foot and ankle problems per rheumatic disorder. In a workshop of 1,5 h participants practiced the use of palpation and ultrasound to detect synovitis in patients with a rheumatic disorder. At the end of the educational programme, the participants practiced the use of the protocol. In a case study presentation, participants practiced with treatment options and evaluating treatment goals. After the day program, participants received a digital link for an e-learning of 30 min on the recognition and diagnoses of skin and nail problems of the foot in rheumatic disorders. Several teachers provided part of the program, including a medical doctor (not specialized), a physician assistant in rheumatology and ultrasound specialist, nurses with a specialization in rheumatology, a podiatrist and the researcher (EHUI). Prior to the educational programme, podiatrists received a digital version of the podiatry protocol, with the request to read it thoroughly.

### Measurements

#### Evaluation of educational programme

The educational programme was evaluated by an anonymous digital questionnaire directly after the day programme. The questionnaire was derived from a similar questionnaire developed by Rooij et al. [27]. The questionnaire in our study consisted of 20 questions evaluating participants’ satisfaction with the different topics of the educational programme. Questions 1–15 concerned the different topics that were addressed in the educational programme (rated as ‘inadequate’, ‘enough’, ‘good’, ‘very good’ or ‘not applicable’). There were three open questions: what did you miss in the programme? Which items of the programme were unnecessary? and do you have any comments? In addition, one question concerned the duration of the educational programme (rated as ‘too short’, ‘good’, ‘too long’ or ‘not applicable’) and one concerned the rating of the educational programme on a 0–10 point scale (with higher score indicating more satisfaction).

#### The use of the podiatry protocol

The protocol was evaluated by an anonymous digital questionnaire after podiatrists used the protocol for three months in their daily practice (from April 2019 to June 2019). The questionnaire was derived from a similar a questionnaire developed by van der Wees et al. [28] on barriers and facilitators for implementing Dutch COPD guidelines for physical therapists in the Netherlands. Our questionnaire consisted of 29 items on facilitators and barriers in using the protocol. Each item was scored on a 5-point Likert scale, ranging from ‘totally disagree’ to ‘totally agree’. Some statements were formulated positively and others negatively.

In addition, to get more in-depth understanding on the barriers and facilitators in the use of the protocol, semi-structured interviews were held. All participants of the pilot were invited per email and telephone. The interviews were conducted by telephone by female lecturer, podiatrist and researcher (EHUI). Her previous experience in qualitative research was limited to qualitative research in training situation. The researcher had a professional relationship in podiatry care with several participating podiatrists, prior to study commencement. However, participants were unaware of personal goals or reasons for doing the research and no characteristics were reported about the interviewer. A topic guide was used to structure the interview (see Additional file 3). Topics were based on the questionnaire on barriers and facilitators, the methodology of implementation by Grol [29] and by process evaluation of implementation as described in Hoekstra et al. [30]. JD and MvdL advised on the topic guide. The duration of the interviews was approximately 30 min.

#### Descriptives

The following characteristics of the participating podiatrists were gathered: age, sex, years of work experience as a podiatrists and years of work experience in a specific rheumatology setting. In addition, the participants were asked to register how many patients with rheumatic foot problems they had treated according to the protocol, how many patients were referred and whether they did not use the protocol in potentially eligible patients.

### Data analysis

#### Evaluation of the educational programme

The number of items scored per topic (‘not applicable’, ‘inadequate’, ‘enough’, ‘good’ or ‘very good’) was converted to a percentage. The researcher (EHUI) reviewed the answers to the three open questions and highlighted relevant topics. Scores on the duration of the educational programme were tabulated as mean (SD). A mean (SD) was also calculated of the marks given for overall satisfaction with the educational programme. The senior research team (JD, MTD, MG and MvdL) advised on the data analysis.

#### Evaluation of the use of the protocol

Descriptive statistics were also used to analyse the digital questionnaires. The ‘disagree’ and ‘strongly disagree’ of the positively formulated questions and the ‘agree’ and ‘strongly agree’ of the negatively formulated questions concern barriers. The ‘agree’ and ‘strongly agree’ of the positively formulated questions and the ‘disagree’ and ‘strongly disagree’ of the negatively formulated questions concern facilitators. The number of completed surveys multiplied by the questions from the category results in a maximum number. A percentage is then calculated for facilitators, barriers and neutral answered topics. Researcher (EHUI) analysed the questionnaires and the senior research team (JD, MTD, MG and MvdL) advised. The interviews were not recorded by the researcher (EHUI). Field notes were made during each interview by the researcher (EHUI). A member-check was done asking the podiatrists whether the interpretation of the researcher (EHUI) was correct, by giving a summary at the end of the interview. All interviews were analysed by the researcher (EHUI) using the steps of thematic analysis [31]. The thematic analysis consisted of six phases: 1) familiarizing with the data, 2) generating initial codes, 3) searching for themes, 4) reviewing themes, 5) defining and naming themes and 6) producing the report [31]. The researcher (EHUI) archived all relevant data. Each file was named to represent the participant from whom the data came. Then the researcher (EHUI) analysed the interviews and coded into categories with similar meaning. The categories with similar meaning were then coded into themes and then refined and defined. Researcher MvdL advised on the coding. When the final themes were established, the COREQ criteria were used for the reporting of the qualitative evaluation [32]. We used the methodological orientation of content analysis, the topic of data saturation seems therefore less relevant [33].

## Results

### Evaluation of the educational programme

A total of 32 podiatrists participated in the educational programme. Despite two reminders, 30 podiatrists evaluated the educational programme by questionnaire as shown in Table 1.

**Table 1:** The percentage of participants scoring a certain response category per question (*n* = 30)

	Not applicable	Inadequate	Enough	Good	Very good
The educational programme was helpful with regard to:					
Basic knowledge about medication use in rheumatic disorders	0%	23%	30%	43%	3%
The latest insights into the treatment of rheumatic disorders	0%	13%	37%	40%	10%
Basic knowledge about the use of ultrasound to detect inflammatory activity in rheumatic disorders	0%	0%	40%	47%	13%
Recognizing foot problems in relation to problems in the knee, hip of back, specifically for rheumatic disorders	3%	10%	43%	40%	3%
Recognizing foot problems with an inflammatory component	0%	0%	43%	47%	10%
Recognizing foot problems with a biomechanical component, specifically for rheumatic foot problems	3%	3%	37%	50%	7%
Recognizing specific rheumatic nail abnormalities	7%	17%	37%	37%	3%
Being able to palpate joints to detect inflammatory activity	0%	3%	30%	50%	17%
Knowledge about the scientific evidence of treatment options for rheumatic foot problems	7%	13%	47%	20%	13%
Using the indication matrix to choose treatment options	0%	13%	40%	30%	17%
Knowing the principles of ‘stepped care’	0%	3%	47%	33%	13%
Knowledge about the criteria for referring to other disciplines	0%	3%	47%	43%	7%
The use of measuring instruments to evaluate treatment goals	3%	7%	50%	33%	7%
Regarding the form of the educational programme, the degree of variety in the programme is:	0%	0%	37%	43%	20%
Regarding the form of the educational programme, the use of practice cases in the programme is:	0%	0%	33%	43%	23%
What did you think of the duration of the educational programme?	33% too short	67% good	0% too long		
On average I give the educational programme the grade: (between 1 and 10)	7.6 (SD1.11)				

The mean satisfaction score with the entire educational programme was 7.6 (SD 1.11). Practical knowledge on joint palpation, variation in the educational programme and the use of practice cases were valued most; > 60% of the podiatrists scored ‘good’ or ‘very good’ on these questions.

A proportion of the participants missed information on medication use and nail disorders; 23 and 17% scored ‘inadequate’ respectively. In the open questions participants stated that the topic of ultrasound was discussed too extensively. With regard to the duration of the programme 33% of all podiatrists thought the programme was too short.

In Table 2 the results of the open questions in the evaluation of the educational programme are presented.

**Table 2:** Open questions in the evaluation of the educational programme

Open questions	Response to the open questions
**Which parts did you miss in the educational programme?**	• Pharmacology, pathologies and connecting with other professions. • In depth understanding of referral criteria. • More rheumatism specific foot symptoms, how to recognize and treat. • More nail and skin disorders. • More opportunity to practice. • How to enter a business relationship with regard to rheumatic foot problems. • Information on the use of the checklists.
**Which parts did you find unnecessary in the educational programme?**	• The use of ultrasound was to extended. • Practicing ultrasound. • The use of the last 2 practice cases. • Clinical reasoning in 2 practices cases instead of 1. • The preparation was too much.
**Do you have any comments regarding the educational programme?**	• The space was too small. • Rather no e-learning. The e-learning is pleasant. • Prefer a list of medication. • The course material is not inherent after one day. • More and elaborated education is needed. • The use of ‘healthy’ patients prior to the use of patients with a rheumatic disorder. • Distinguish between basic screening and extensive screening. • Skills require more training. Dosed pace for more in depth understanding.

### Evaluation of the use of the protocol in clinical practice

During the pilot phase four of the 32 podiatrists dropped out for personal reasons. One podiatrist could not be reached, despite several attempts. A total of 27 podiatrists evaluated the use of the podiatry protocol in clinical practice. The characteristics of the participants are shown in Table 3.

**Table 3:** Pilot phase participant characteristics (*N* = 27)

Characteristics	Value
Female	85%
Age, mean (SD, range) years	39 (11.2; 24–61)
Work experience, mean (SD, range) years	11 (8.6; 2,5–35)
Experience in a specific rheumatology setting^a^ N=	4

^a^ Defined as: a specific rheumatology setting such as a rheumatology department or a long-term collaboration with a rheumatologist

Despite several reminders, a total of 23 out of 28 questionnaires were completed at the end of the pilot phase. During the three months of the pilot testing, 27 podiatrists treated a total of 193 patients with rheumatic foot problems according to the protocol. Podiatrists were not asked in how many patients with foot problems in rheumatic disorders they did not use the protocol. Of the 193 treated patients, 114 were referred by a medical doctor, the others came directly to the podiatrists without referral. In Table 4 the characteristics of the treated patients are shown. Three podiatrists had not seen any patients with rheumatic foot problems or did not use the protocol in these patients. Two podiatrists stated that they also used the protocol for patients with scleroderma and fibromyalgia. Also, two podiatrists had not scored the amount of patients they had treated according to the protocol or could not remember exactly. These numbers were therefore not included.

**Table 4:** Patients with a rheumatic disorder and foot problems treated by podiatrists using the protocol in clinical practice (*N* = 193)

Characteristics	N
Patients treated per podiatrist average	7.4
Total treated patients with a rheumatic disorder	193
RA	52
OA	84
(pseudo) Gout	43
SpA	8
Unfamiliar	6
Already diagnosed with a rheumatic disorder	157
Referred by medical doctor	114

Facilitators and barriers were identified, as shown in Table 5.

**Table 5:** The percentage of participants scoring a certain response category on questions related to facilitators and barriers for the use of the podiatry protocol for rheumatic foot problems (*n* = 23)

	(Strongly) Disagree	Neither agree nor disagree	(Strongly) Agree
Facilitators			
The use of the protocol supports me in which rheumatism-related foot symptoms I have to monitor	9%	4%	87%
The recommendations on the treatment are clear and understandable to me	4%	9%	87%
The use of the protocol helps me to improve my knowledge	26%	13%	78%
The use of the protocol supports me in using the diagnostic categories	4%	22%	74%
The use of the protocol supports me in the use of ‘stepped care’ treatment	9%	17%	74%
I have read or remembered the instruction to apply it	13%	13%	74%
In the practice where I work is sufficient equipment to apply it	4%	22%	74%
Patients cooperate well in applying it	0%	26%	74%
The use of the protocol supports me in clinical reasoning	4%	26%	70%
Adjustments in the diagnostic phase are clear and understandable for me	4%	35%	61%
I have enough skills to apply it	17%	22%	61%
The protocol is easy to use	22%	22%	57%
I have enough knowledge to apply it	26%	26%	48%
The protocol is applicable in daily practice	26%	30%	44%
Managers work well in applying it	4%	52%	44%
The protocol fits my way of working	44%	17%	39%
I can integrate it well into daily activities	26%	44%	30%
The lay-out promotes ease of use	30%	35%	35%
The protocol invites me to consult more with experts	26%	39%	35%
I see enough patients with rheumatic foot problems to be able to apply it	39%	30%	30%
Colleague podiatrists work well in applying it	13%	65%	22%
GP’s or other specialists work well in applying it	13%	83%	4%
Barriers			
It takes too much time	4%	30%	65%
There should be a financial compensation for working with it	26%	30%	44%
The reimbursement for the treatment that the patients receives is an obstacle to apply it	30%	48%	22%
The protocol leaves no room for my own decisions	57%	35%	9%
I first use other therapies before I switch to inlays	30%	61%	9%
I generally have resistance to working with treatment protocols	61%	30%	9%
I think that certain parts are incorrect	61%	35%	4%

Two main facilitators were identified: 1) the protocol supports patient management in patients with foot symptoms related to rheumatic disorders and 2) the recommendations on the treatment are clear and understandable. Three main barriers were identified: 1) the use of the protocol takes time, 2) the current financial compensation is an obstacle to apply the protocol and 3) the reimbursement for the treatment that the patients receives is an obstacle to apply the protocol.

A total of 27 semi-structured telephone interviews were completed. Six themes were identified: 1) time, 2) scope of the underlying documents, 3) integration in the patient file, 4) professional practice and 5) implementation.

The results of the interviews reflect the results of the questionnaires. The time the use of the protocol takes, was identified as one of the three most important barriers. The interviews showed that there is a relation between the amount of time (theme 1) the protocol took and the scope of the underlying documents of the protocol (theme 2). The provided documents of the protocol were developed as a tool. However, participants stated that they used the documents to explicitly fill in and explain each item. Therefore, it took time to use the protocol: “The list is too long and then it becomes a big task to do” said participant 18. The amount of time it takes is also related to the integration of the protocol in the patient file. In the Netherlands podiatrist use specific software (a digital patient file) in which they register the medical data of patients. There are several software programs available for podiatrists and usually they have specific entry fields for specific foot problems to facilitate and speed up the filling in. Because the protocol has not been integrated in the patient file (theme 3), podiatrist stated that it took them more time. “It might already matter if it is in the patient file” (participant 23). ‘Professional practice’ (theme 4) is related to the two main facilitators: ‘the support of the protocol during patient management’ and ‘the clear and understandable treatment recommendations’. The protocol seemed to help them in their professional practice. It improved their knowledge and skills: “Especially the educational programme has influenced my knowledge and skills” (participant 1). The theme ‘implementation’ (theme 5) refers to the points of attention in implementing of the protocol. It seems podiatry care is paramount in rheumatic foot problems: “It must be made clear that podiatrists have something to say about rheumatology” (participant 3).

The two barriers: ‘financial compensation’ and ‘reimbursement for the treatment’ were discussed less extensively in the interviews. ‘Reimbursement for the treatment’ concerns podiatric care for patients with a rheumatic disorder in general. In the Netherlands, there is no reimbursement for the treatment from the basic health insurance package for podiatric care for rheumatic disorders. It is, however, possible to take out additional insurance. Participant 7 said: “all my patients are additionally insured. But I can imagine that the lack of reimbursement for the treatment could play a role in the application of the protocol”. The barrier ‘financial compensation’ seems to be related to the topic of time. Podiatrists stated that the more time the protocol takes, the more money it costs. “More money is needed to arrange more time” stated participant 9.

## Discussion

The results of this study show that an educational programme is helpful in the use of the clinical protocol for podiatry management of foot problems in rheumatic disorders. The main facilitators in the use of the protocol were related to the support during patient management and to the clear and understandable treatment recommendations. Main barriers to using the protocol are related to the amount of time, the current financial compensation and the reimbursement for the treatment that the patients receives. These results should be taken into account when implementing the protocol large-scale.

The mean satisfaction with the educational programme was 7.6. However, information on medication use and nail disorders was missed and the ultrasound topic was found to be too extensively discussed. Scientific developments in medication for rheumatic diseases are rapid [30]. We therefore recommend to adjust the educational programme and to pay more attention to medication, as well as on nail disorders. Ultrasound for foot problems in rheumatic disorders could be a separate educational programme.

The use of the protocol, in particular the use of the checklists, took more time than expected. Mainly because the protocol has not yet been implemented in the patient files. Time was also experienced as a barrier in the study of Williams et al. [21] on the use of clinical guidelines in the management of foot health problems related to rheumatoid arthritis in the United Kingdom. In that study podiatrists indicated that there is a lack of time in clinical practice for reading clinical guidelines. However, in the study of Landsdowne et al. [34] on barriers in the management of foot health in patients with a rheumatic condition, a positive attitude was shown, which they believe may help to overcome the barrier of time. Despite the fact that the podiatrists in our study indicated that the protocol takes time, they also indicated that the protocol was useful and has added value. We recommend podiatrists to integrate the protocol into the electronic patient record system to avoid duplication of work and to use only those parts of the protocol that are applicable to the patient in question.

The barrier of financial compensation experienced by the podiatrists seems to be related to the time required by the protocol. Financial compensation was also experienced as a barrier in the study on the implementation of diabetes guidelines in the Netherlands [35]. However, If the time required by the protocol can be reduced, by using it as a tool and implementing it in the patient file, the financial compensation could be a barrier to less extent.

Our study also revealed that the reimbursement for the treatment received by the patients is an obstacle in applying the protocol. However, this barrier is not specific to the protocol but to the reimbursement of podiatric care for rheumatic diseases in general. The reimbursement for podiatric care for patients with a rheumatic disorder in the Netherlands comes from the supplementary healthcare insurance package. A similar barrier was found in a study of McCulloch et al. [36] on the experiences of podiatrists on the foot care for people with arthritis in the United Kingdom. Podiatrists stated that podiatry care varied throughout the healthcare systems. Podiatry service might not be accessible for every patient in need of foot care. Causing an inequality of foot care.

The results of our pilot show that the podiatrists found the protocol useful and an added value. There were no inaccuracies in the protocol according to the podiatrists. They thought the protocol was an important tool. It helped them to improve their knowledge and skills. Specifically, the educational programme was deemed helpful. The importance of education is also seen in a study of Carter et al. [37] on health professionals’ view on the assessment and management of patients with psoriatic arthritis and foot problems. Health professionals in rheumatology, such as the podiatrist, stated a lack of appropriate training and expertise for the management and treatment of foot problems in patients with psoriatic arthritis. Future research should be undertaken to investigate the quality and outcomes of foot care delivered according to the protocol, to determine whether better results are gained for the input of more time.

This pilot study has some limitations. Seven of the 11 participants of the consensus meeting also participated in the evaluation of the protocol. It is possible that these participants may therefore be biased, because they were involved in the development of the protocol. There was some drop-out during the study. Not every podiatrist who participated in the educational programme tested the protocol and not every participating podiatrist filled in the digital questionnaires or participated in the interviews. We did not define an acceptable level of drop-out in advance. However, the Scottish Intercollegiate Guidelines Network (SIGN) published a methodology checklist for cohort studies in which they state that a drop-out level of 20% is considered acceptable [38]. In our study 5 of the 28 participants dropped-out, resulting in a 18% drop-out level, which therefore can be considered acceptable. Another limitation of our study is that one researcher (EHUI) developed the educational programme, did the interviews and analysed the data. This may have affected participants’ responses, thereby introducing bias. To minimize bias, interviews were held by telephone instead of face to face, questionnaires were anonymous and the senior research team (JD, MTD, MG and MvdL) advised on the data acquisition and data analysis.

A strength of this study is the representativeness of the participating podiatrists in terms of validity. The majority of Dutch podiatrists is female and work in a private practice, as the participating podiatrists do. However, no in-service training for podiatrists directed to foot problems in rheumatic disorders exists in the Netherlands. Their experience with rheumatology therefore depends on personal interest and motivation. Because this was a motivated group of participants that has signed up to participate, the results may not be generalizable to the total group of podiatrists. Another strength of this study is the combination qualitative and quantitative strategies to achieve triangulation. This “methods triangulation” [39] was used to enhance reliability [40].

## Conclusions

The educational programme concerning the systematic podiatry protocol for foot problems in rheumatic disorders appears to be helpful for podiatrists. Podiatrists perceived the protocol as being supportive during patient management. Barriers for use of the protocol in podiatry practice were identified and should be addressed prior to large scale implementation. Whether the protocol is also beneficial for patients, needs to be determined in future research.

## Supplementary Information

**Additional file 1.** Confirmation letter Medical Ethics Review Committee of VU University Medical Centre.

**Additional file 2.** Development of the systematic podiatry protocol.

**Additional file 3.** Topic guide.

## Data Availability

The datasets during and/or analysed during the current study are available from the corresponding author on reasonable request.

## Abbreviations

NVvP: Dutch Association of Podiatrists
OA: Osteoarthritis
RA: Rheumatoid arthritis
SD: Standard deviation
SpA: Spondyloarthritis
WMO: Medical Research Involving Human Subjects Act
