# Correction to: Clinical guidance for podiatrists in the management of foot problems in rheumatic disorders: evaluation of an educational programme for podiatrists using a mixed methods design

**DOI:** 10.1186/s13047-021-00463-x

**Published:** 2021-05-06

**Authors:** E. J. Huijbrechts, J. Dekker, M. Tenten-Diepenmaat, M. Gerritsen, M. van der Leeden

**Affiliations:** 1 Amsterdam Rehabilitation Research Centre | Reade, Dr. Jan van Breemenstraat 2, PO 58271, 1040, HG Amsterdam, The Netherlands; 2 Fontys University of applied sciences | Department of allied health professionals, Fontys Paramedische Hogeschool, Eindhoven, The Netherlands; 3 Department of Rehabilitation Medicine, Amsterdam UMC, Vrije Universiteit van Amsterdam, Amsterdam, The Netherlands; 4 Saxion University of Applied Sciences | Department of Healthcare, Saxion, Enschede, The Netherlands

**Correction to: J Foot Ankle Res 14, 15 (2021)**

**https://doi.org/10.1186/s13047-020-00435-7**

Following publication of the original article [1], we were notified that Fig. 2 should be the figure of the supplementary material (Additional file 2). The correct Fig. 2 has been placed as an Additional file 4.

The original article has been corrected. Additional file 2 has been replaced.

## Supplementary Information

**Additional file 2.** Development of the systematic podiatry protocol.
