# Supplementary material for: Clinical guidance for podiatrists in the management of foot problems in rheumatic disorders: evaluation of an educational programme for podiatrists using a mixed methods design
Source: J Foot Ankle Res. 2021 Feb 25;14:15. doi: 10.1186/s13047-020-00435-7 (PMC7908782; doi:10.1186/s13047-020-00435-7)
Supplement: Supplementary file 1 — Additional file 1. Confirmation letter Medical Ethics Review Committee of VU University Medical Centre. [file 13047_2020_435_MOESM1_ESM.pdf]

Amsterdam UMC
Universitair Medische Centra

M. van der Leeden
afdeling revalidatie geneeskunde
PK -1Y 158

Medisch Ethische Toetsingscommissie VUmc
Van der Boechorststraat 7, kamer H-443
Postbus 7057
1007 MB Amsterdam
020 444 5585
www.vumc.nl/METc

Datum: 23 januari 2019
Ons kenmerk: 2019.010
Betreft: Niet-WMO advies

Geachte mevrouw van der Leeden,

Het Dagelijks Bestuur van de Medisch Ethische Toetsingscommissie VU medisch centrum heeft uw onderzoek **Methodisch podotherapeutisch handelen bij reumatische aandoeningen: evaluatie van de toepasbaarheid in de podotherapeutische praktijk.** besproken in de vergadering van 22/01/2019.

Het onderzoek valt niet onder de reikwijdte van de Wet Medisch-wetenschappelijk Onderzoek met mensen (WMO).

Het oordeel is gebaseerd op de volgende documenten:

Sectie	Onderwerp	Versie
A1	aanbiedingsbrief	d.d. 10-1-2019
A1	correspondentie	d.d. 23-1-2019
C1	onderzoeksprotocol	versie 1 d.d. 23-12-2018
E11	informatiebrief	versie 2 d.d. 10-1-2019
E2	toestemmingsverklaring	versie 2 d.d. 10-1-2019
F1	vragenlijst	evaluatie scholing MPH, evaluatie toepasbaarheid in praktijk en interview

Het Dagelijks Bestuur van de Medisch Ethische Toetsingscommissie VU medisch centrum wijst u erop dat hoewel het ingediende onderzoek niet onder de reikwijdte van de WMO valt, andere wet- en regelgeving (mogelijk) wel van toepassing is, waaronder:

- WGBO (Wet Geneeskundige BehandelingsOvereenkomst);
- AVG (Algemene Verordening Gegevensbescherming), zie https://autoriteitpersoonsgegevens.nl/nl/onderwerpen/avg-europese-privacywetgeving;

AMC en VUmc werken samen in Amsterdam UMC

VUmc
De Boelelaan 1117
1081 HV Amsterdam
Postbus 7057
1007 MB Amsterdam
T +31(0)20 444 4444
www.vumc.nl

- Code Goed Gedrag (Gedragscode gezondheidsonderzoek: gebruik medische gegevens in wetenschappelijk onderzoek), zie www.federa.org;
- Code Goed Gebruik (Gedragscode Verantwoord omgaan met lichaamsmateriaal ten behoeve van wetenschappelijk onderzoek, 2011), zie www.federa.org;
- Biobanken: Reglement toetsing biobank VUmc, zie https://www.vumc.nl/afdelingen/METc/biobank/;
- WBO (Wet Bevolkings Onderzoek), zie http://www.vumc.nl/afdelingen/METc/wetgeving/wetbevolkingsonderzoek/.

To whom it may concern

We are pleased to confirm that the Medical Research Involving Human Subjects Act (WMO) does not apply to the above mentioned study and that an official approval of this study by our committee is not required.

The Medical Ethics Review Committee of VU University Medical Center is registered with the US Office for Human Research Protections (OHRP) as IRB00002991. The FWA number assigned to VU University Medical Center is FWA00017598.

Met vriendelijke groet,
namens de Medisch Ethische Toetsingscommissie VU medisch centrum,

p.o.

prof. dr. J.A.M. van der Post, voorzitter

c.c.: afdelingshoofd Revalidatie Geneeskunde prof. dr. V. de Groot

AMC en VUmc werken samen in Amsterdam UMC

VUmc
De Boelelaan 1117
1081 HV Amsterdam

Postbus 7057
1007 MB Amsterdam

T +31(0)20 444 4444
www.vumc.nl
