# Supplementary material for: Clinical guidance for podiatrists in the management of foot problems in rheumatic disorders: evaluation of an educational programme for podiatrists using a mixed methods design
Source: J Foot Ankle Res. 2021 Feb 25;14:15. doi: 10.1186/s13047-020-00435-7 (PMC7908782; doi:10.1186/s13047-020-00435-7)
Supplement: Supplementary file 3 — Additional file 3. Topic guide. [file 13047_2020_435_MOESM3_ESM.docx]

**Additional file III Topic guide**

- General
  - Personal characteristics (1)
  - Age (1)
  - Work experience (1)
  - Membership (1)
- Applicability
  - General applicability
  - Integration
  - Clinical reasoning
  - Contribution
  - Inaccuracies
  - Distinctness
  - Lay-out
  - Equipment
  - Competence (1)
  - Willingness (1)
  - Effects
  - Efficacy (2)
  - Range (2)
- Education
  - Preparation
  - Learning style (1)
  - Self-efficacy
  - Adoption (2)
- Coaching
  - Adoption (2)
  - Implementation (2)

**References**

1. Grol R. Implementing guidelines in general practice care. Quality in health care : QHC. 1992;1(3):184-91.

2. Hoekstra F, Alingh R, Schans vdCP, Hettinga FJ, Duijff M, Dekker R, et al. Design of a process evaluation of the implementation of a physical activity and sports stimulation programme in Dutch rehabilitation setting: ReSpAct. Implementation science. 2014;9(1):127.
