# Supplementary material for: Correction to: Clinical guidance for podiatrists in the management of foot problems in rheumatic disorders: evaluation of an educational programme for podiatrists using a mixed methods design
Source: J Foot Ankle Res. 2021 May 6;14:37. doi: 10.1186/s13047-021-00463-x (PMC8101093; doi:10.1186/s13047-021-00463-x)
Supplement: Supplementary file 1 — Additional file 2. Development of the systematic podiatry protocol. [file 13047_2021_463_MOESM1_ESM.pdf]

Additional file II Development of the systematic podiatry protocol

The protocol is based on a Dutch explanation of podiatric reporting according to the steps of methodical action. The Dutch explanation of podiatric reporting has been written by The Dutch Association of Podiatrists (NVvP). It describes the steps taken to report on foot problems. However, this is not a guideline or treatment protocol. It is not based on the best available evidence and it is not specific to rheumatic foot problems.

Several steps were taken to develop the clinical guidance protocol for podiatrists in the management of foot problems in rheumatic disorders. The Medical Research Council (MRC) framework of developing a complex intervention were used (1). See also Figure Ia.

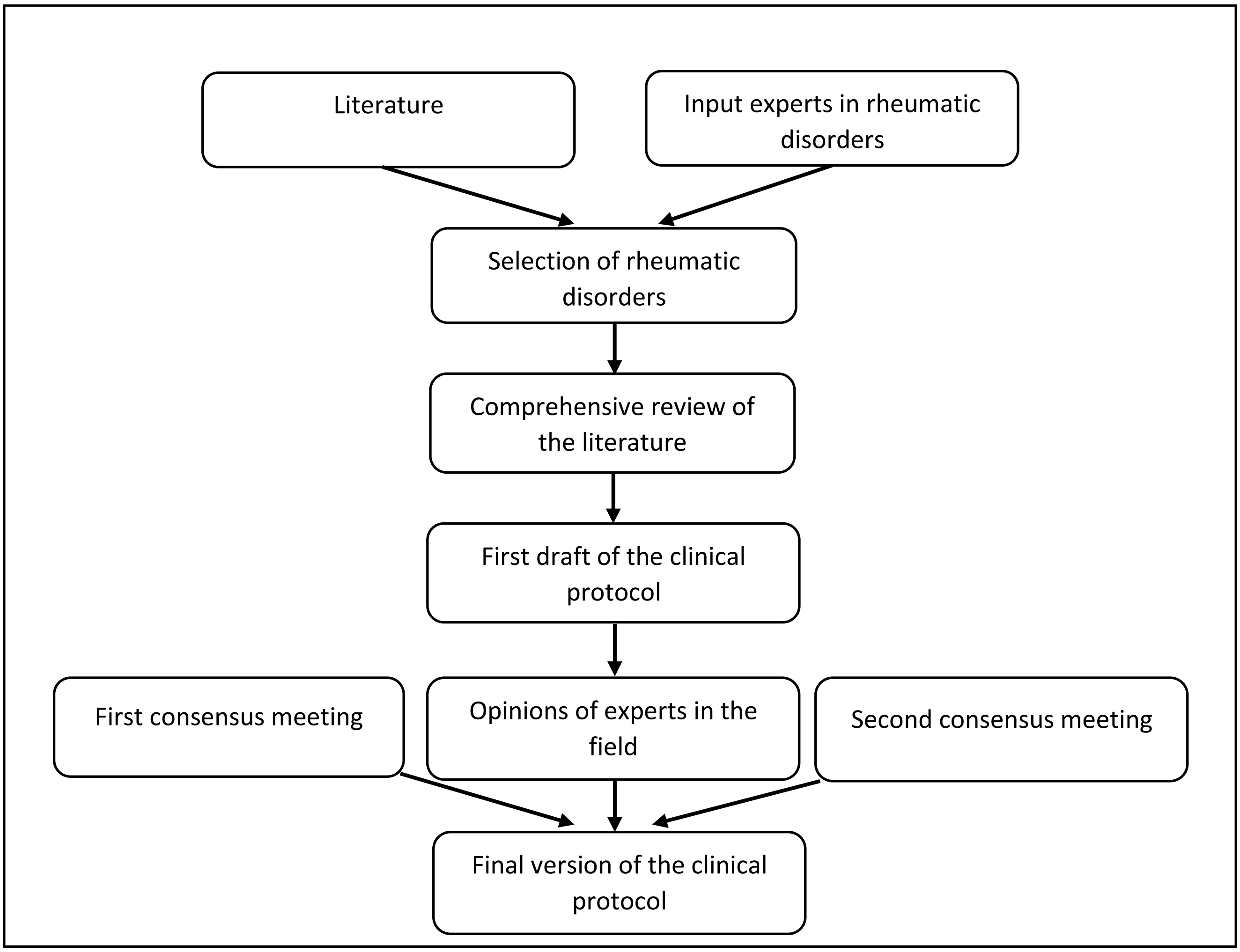

Figure Ia Development of the systematic podiatry protocol in the management of foot problems in rheumatic disorders.

First, a selection of rheumatic disorders was made based on previous work (2), literature and opinion of experts in the field of rheumatology. The following rheumatic disorders were selected: rheumatoid arthritis (RA), spondyloarthritis (SpA), (pseudo)gout and osteoarthritis (OA). Second, a systematic literature search was conducted in order to identify diagnostic categories of foot problems in rheumatic disorders. Third, a preliminary version of the protocol was developed based on the first two steps, consultation of international guidelines for foot health (3, 4) and podiatry books (5-7). Fourth, the preliminary version of the protocol was discussed in a consensus meeting. Also, individual experts in the field were asked their opinion of the protocol. The experts had extensive experience in the field of foot problems, rheumatology or both. After optimizing the protocol, a second consensus meeting was held with podiatrists for the collection of feedback and to gain consensus on the protocol. Resulting in a clinical guidance protocol for podiatrists. The care provided is not new however, the protocol systematically guides podiatrists in the use of the multidisciplinary recommendations (2).

There are many factors associated with foot problems in these four rheumatic disorders. A classification of those factors is currently not possible of several reasons. Firstly, rheumatic foot problems show a variety of symptoms. Secondly, the severity of rheumatic foot problems differs per patient. Thirdly, evidence for causal relationships between factors and rheumatic foot problems is lacking. Therefore, an indication matrix (see also Table 1) was developed of four rheumatic disorders and six diagnostic categories of rheumatic foot problems, instead of a classification.

Table 1 Indication matrix of four rheumatic disorders (rheumatoid arthritis (RA), Spondyloarthritis (SpA), gout and osteoarthritis (OA)) and six diagnostic categories. One cell in the table is marked black because no evidence had been found in the literature that dermatological abnormalities occur in osteoarthritis.

Rheumatic disorder	Diagnostic categories					
	Active inflammation of foot structures	Abnormal foot position and foot function pathologies	Dermatological pathologies	Peripheral neurovascular pathologies	Inadequate shoes	Different expectations about the treatment
RA						
SpA						
Gout						
OA						

The indication matrix has been used to shape the process of management (history taking, physical examination, treatment and evaluation). A full version of the protocol can be found in Figure Ib.

Figure Ib Full version of the systematic podiatry approach for foot problems in rheumatic disorders

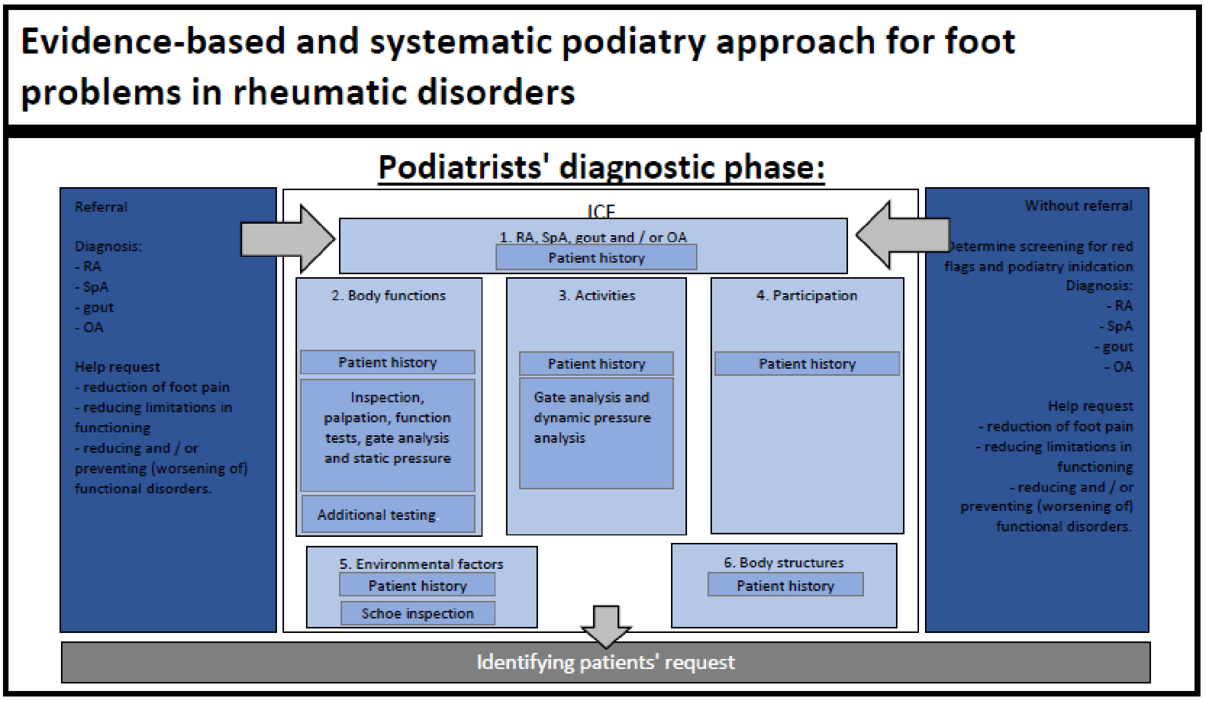

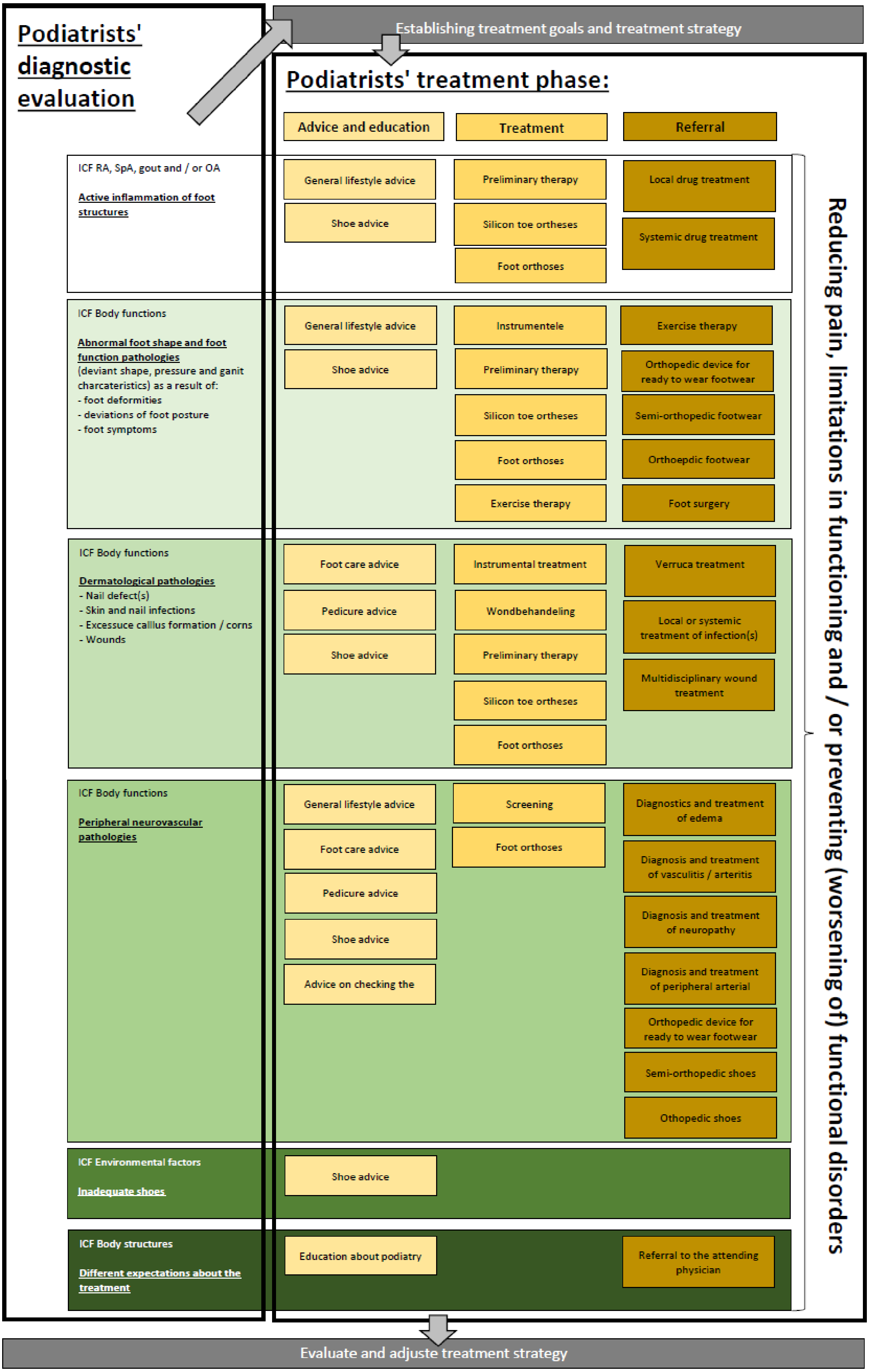

Podiatrists' diagnostic evaluation
Establishing treatment goals and treatment strategy
Podiatrists' treatment phase:
Advice and education
Treatment
Referral
ICF RA, SpA, gout and / or OA
Active inflammation of foot structures
General lifestyle advice
Shoe advice
Preliminary therapy
Silicon toe ortheses
Foot orthoses
Local drug treatment
Systemic drug treatment
ICF Body functions
Abnormal foot shape and foot function pathologies
(deviant shape, pressure and ganit charcateristics) as a result of:
- foot deformities
- deviations of foot posture
- foot symptoms
General lifestyle advice
Shoe advice
Instrumentele
Preliminary therapy
Silicon toe ortheses
Foot orthoses
Exercise therapy
Exercise therapy
Orthopedic device for ready to wear footwear
Semi-orthopedic footwear
Orthoepdic footwear
Foot surgery
ICF Body functions
Dermatological pathologies
- Nail defect(s)
- Skin and nail infections
- Excessuce callus formation / corns
- Wounds
Foot care advice
Pedicure advice
Shoe advice
Instrumental treatment
Wondbehandeling
Preliminary therapy
Silicon toe ortheses
Foot orthoses
Verruca treatment
Local or systemic treatment of infection(s)
Multidisciplinary wound treatment
ICF Body functions
Peripheral neurovascular pathologies
General lifestyle advice
Foot care advice
Pedicure advice
Shoe advice
Advice on checking the
Screening
Foot orthoses
Diagnostics and treatment of edema
Diagnosis and treatment of vasculitis / arteritis
Diagnosis and treatment of neuropathy
Diagnosis and treatment of peripheral arterial
Orthopedic device for ready to wear footwear
Semi-orthopedic shoes
Othopedic shoes
ICF Environmental factors
Inadequate shoes
Shoe advice
ICF Body structures
Different expectations about the treatment
Education about podiatry
Referral to the attending physician
Reducing pain, limitations in functioning and / or preventing (worsening of) functional disorders
Evaluate and adjuste treatment strategy

References

1. Craig P, Dieppe P, Macintyre S, Michie S, Nazareth I, Petticrew M. Developing and evaluating complex interventions: new guidance. In: MedicalResearchCouncil, editor. Received on 17-09-2018 by https://mrc.ukri.org/documents/pdf/complex-interventions-guidance/
2. Tenten-Diepenmaat M, van der Leeden M, Vliet Vlieland TPM, Dekker J, on behalf of the RAFEG. Multidisciplinary recommendations for diagnosis and treatment of foot problems in people with rheumatoid arthritis. Journal of Foot and Ankle Research. 2018;11(1).
3. Williams AE, Davies S, Graham A, Dagg A, Longrigg K, Lyons C, et al. Guidelines for the management of the foot health problems associated with rheumatoid arthritis. Musculoskeletal Care. 2011;9(2):86-92.
4. PodiatryRheumaticCareAssociation. Standards of care for people with musculoskeletal foot health problems. London: Podiatry Rheumatic Care Association. 2008.
5. Helliwell P, Woodburn J, Redmond A, Turner D, Davys H. The foot and ankle in rheumatoid arthritis: a comprehensive guide. London: Elsevier Health Sciences; 2007.
6. Frowen P, O'Donnell M, Lorimer D, Burrow JG. Neale's disorders of the foot. 6 ed. Churchill Livingstone: Elsevier Health Sciences; 2010.
7. van Putten M, Huijbrechts E. Voeten en reuma. Houten: Bohn Stafleu van Loghum; 2020.
